# The role of gadoxetic acid-enhanced magnetic resonance cholangiography in the evaluation of postoperative bile duct injury: pictorial essay

**DOI:** 10.1590/0100-3984.2018.0089

**Published:** 2019

**Authors:** Bruno Jucá Ribeiro, Aldo Maurici Araújo Alves, Rafael Santiago de Oliveira, Fernanda Velloni, Giuseppe D'Ippolito

**Affiliations:** 1 Escola Paulista de Medicina da Universidade Federal de São Paulo (EPM-Unifesp), São Paulo, SP, Brazil.

**Keywords:** Iatrogenic disease, Bile ducts, Magnetic resonance imaging, Contrast media

## Abstract

Iatrogenic lesion of the bile ducts is a relatively common occurrence during liver surgery, increasing morbidity and mortality rates. T2-weighted magnetic resonance cholangiography and gadoxetic acid-enhanced functional magnetic resonance cholangiography (fMRC) with administration of hepatobiliary-specific contrast medium (gadoxetic acid) are fundamental to the diagnostic imaging approach in patients with such lesions. Here, we present a review of the literature and suggest an imaging approach to biliary tract injury, focusing on clinical cases in which fMRC had an impact on the decision-making process for the management of the affected patients.

## INTRODUCTION

Iatrogenic lesion of the bile duct is a relatively common occurrence during liver surgery, increasing morbidity and mortality rates^(1)^. Biliary complications such as fistulas and stenoses occur after cholecystectomy in up to 0.6% of cases^(2)^. In addition, patients submitted to biliary-enteric anastomosis are more likely to present complications (e.g., cholangitis and stones), which are estimated to occur in 10-30% of such patients^(3,4)^.

Laboratory tests or imaging examinations can be used in order to evaluate patients suspected of having an iatrogenic lesion of the biliary tract. Laboratory tests, notably quantification of alkaline phosphatase and gamma-glutamyl transferase, are sensitive but not very specific in the characterization of complications^(5)^.

As a general rule, ultrasound and computed tomography do not provide the necessary detail to define the appropriate treatment, being limited to the detection of bile duct dilatation and perihepatic fluid collections^(1)^.

The main disadvantages of endoscopic retrograde cholangiopancreatography (ERCP) and intraoperative cholangiography are their invasiveness, the difficulty in accessing the biliary tract in the presence of biliary-enteric anastomosis, and the high risk of complications, such as intraluminal bleeding and sepsis, as well as the difficulty to use ERCP in performing follow-up evaluations^(5)^. Therefore, magnetic resonance cholangiography (MRC) was established as an effective, noninvasive method for the detection of postoperative complications. By means of T2-weighted MRC (T2-MRC), it is possible to characterize stenoses and dilatations of the bile ducts, although T2-MRC does not provide functional information related to bile excretion and flow, which hinders the differentiation between mild and obstructive dilatation and the characterization of lesions in cases without dilatation^(6,7)^.

T1-weighted MRC with hepatobiliary-specific contrast (gadoxetic acid), also known as functional MRC (fMRC), is an emerging technique that has already proved useful in delineating the preoperative and postoperative anatomy of the biliary tree, as well as in detecting/characterizing biliary diseases and postoperative complications such as intraductal stones, stenoses, biliary fistulas, and cysts^(8,9)^. In addition, it can provide functional information, which is useful for grading biliary stenosis, evaluating the dynamics of bile flow, and estimating the segmental liver function^(10)^. Various studies have demonstrated the usefulness of fMRC-based information on bile flow dynamics, some reporting rates of accuracy in the detection of postoperative lesions even higher than those obtained with T2-MRC^(1)^.

The objective of this essay was to present our experience with fMRC in the context of patients suspected of having postoperative complications of the bile ducts.

## PHARMACOKINETICS AND EXAMINATION PROTOCOL

Gadoxetic acid is an ionic hepatobiliary-specific contrast agent composed of gadolinium and ethoxybenzyl-diethylenetriamine-pentaacetic acid (Gd-EOB-DTPA, Primovist; Bayer Schering Pharma, Berlin, Germany). In addition to the specific extracellular properties of the DTPA component, which allows image acquisition in the arterial, portal, and equilibrium phases, gadoxetic acid has lipophilic properties, provided by the EOB, which allows its gradual uptake (≤ 50% of the injected dose) by normally functioning hepatocytes. During the hepatobiliary phase, which begins, on average, 10-20 min after contrast injection in healthy individuals, it is possible to observe excretion of the contrast medium through the bile ducts, which provides enhancement of the biliary tree on T1-weighted sequences^(10,11)^.

**Figura 1:**
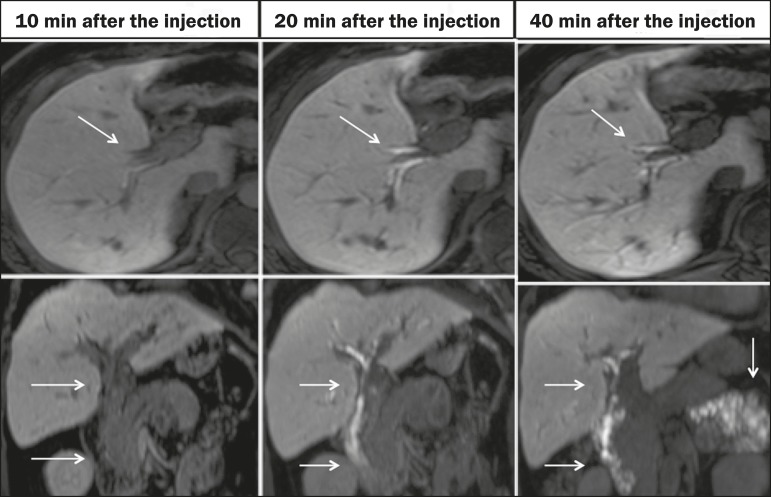
Representation of the normal time for excretion of contrast (gadoxetic acid) through the bile ducts in fMRC: 10, 20, and 40 min after the injection.

**Figura 2:**
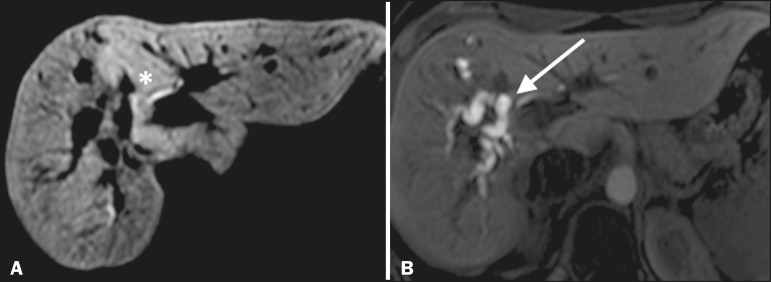
Stenosis of the intrahepatic biliary tract after cholecystectomy. Axial T1-weighted fMRC sequences acquired at 20 min (**A**) and 4 h (**B**) after the injection of gadoxetic acid, showing moderate dilatation of the right biliary tract caused by stenosis in the plane of the right bile duct (arrow in **B**). Note that the concentration of contrast medium is higher in the parenchyma of the left hepatic lobe (asterisk in **A**) than in that of the right lobe, where there is a delay in the enhancement of the bile ducts (**B**). Diagnoses of thermal injury and bile duct ligation were considered.

The half-life of gadoxetic acid is approximately 2 h, the peak of accumulation in hepatocytes occurring at 20-40 min, hepatocyte uptake beginning at 3 min, and biliary excretion beginning at 10 min (Figure 1). High levels of bilirubin (> 3 mg/dL) and ferritin (i.e., hepatic dysfunction) can reduce contrast uptake by the liver, thereby delaying or reducing biliary excretion (Figure 2). Therefore, delayed image acquisition (at 20-180 min, in our experience) or a double dose of the contrast medium might be needed in order to achieve adequate enhancement of the biliary tree^(10,11)^.

The fMRC protocol consists of three-dimensional T1-weighted sequences with fat saturation, axial and coronal images being acquired in the hepatobiliary phase (at 10, 20, and 40 min after injection of the contrast medium). Occasionally, it can be necessary to acquire images even 8-24 h later. At our facility, fMRC examinations are performed in one of two types of 3.0 T scanners: Achieva 3.0 T TX (Philips Medical Systems, Best, The Netherlands), with a repetition time of 1.83 ms, echo time of 3.89 ms, slice thickness of 3 mm, flip angle of 30º, duration of 18 s, and a matrix of 256 × 134-or Skyra 3.0 T (Siemens Healthcare, Erlangen, Germany), with a repetition time of 6.0 ms, echo time of 2.46 ms, slice thickness of 3 mm, flip angle of 30º, duration of 18 s, and a matrix of 256 × 128. The fMRC examinations are usually complemented with traditional sequences of the upper abdomen and T2-MRC.

## INDICATIONS

### Stenosis

**Figura 3:**
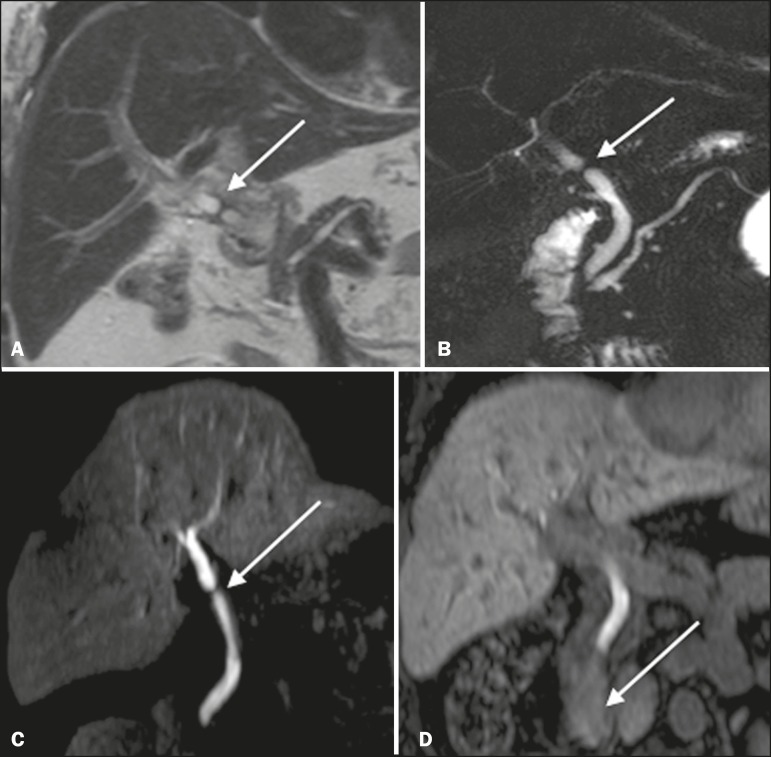
Stenosis of the biliary anastomosis after liver transplantation. Coronal T2-MRC and T1-weighted MRC (**A** and **B**, respectively): stenosis of the biliary anastomosis with mild dilatation of the intrahepatic biliary tree. Coronal fMRC at 40 min after the injection of gadoxetic acid (**C,D**): stenosis (**C**) and a delay in the progression of contrast to the duodenal arc (**D**). Obvious stenosis with a delay in the progression of the contrast medium through the biliary tract.

**Figura 4:**
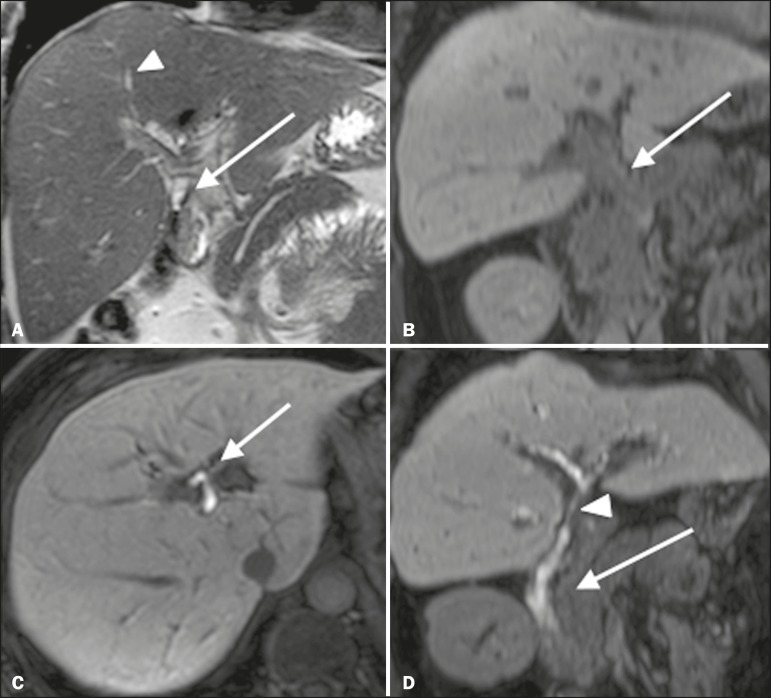
Evaluation of biliaryenteric anastomosis (after complicated cholecystectomy). Coronal T2-weighted sequence (**A**), and T1- weighted fMRC sequences—axial (**C**) and coronal (**B,D**): biliary-enteric anastomosis and mild dilatation of the intrahepatic bile duct 10 min after injection of the contrast medium (arrow and arrowhead, respectively, in **A**), and delayed excretion of contrast (**B**), which was not observed until 20 min after injection of the contrast medium (**C,D**). Patent stenosis of the biliary-enteric anastomosis with delayed biliary excretion.

**Figura 5:**
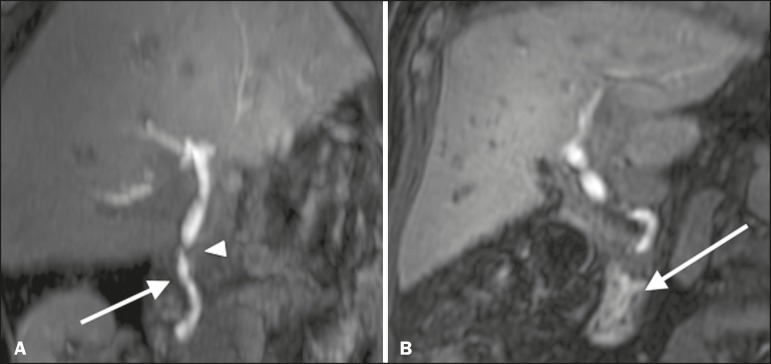
Stenosis of the biliary anastomosis after liver transplantation. Coronal T1-weighted fMRC (**A,B**) with normal progression of contrast medium in the distal bile duct (arrow in **A**) and in the duodenal arc (arrow in **B**). The anastomosis remained patent, despite the narrowing in the plane of the anastomosis (arrowhead).

T2-MRC has limitations in distinguishing the expected, slight patent narrowing in the plane of the biliary anastomosis, without clinical significance, from clinically relevant stenosis. In fMRC, gadoxetic acid provides functional information on biliary excretion in such a way that a delay in the progression of the contrast medium at the suspected site is an important marker of significant stenosis, as is the absence of such progression. In our experience, as well as in other reports in the literature, the time to contrast filling of the biliary tract is directly proportional to the degree of stenosis, and acquisitions at up to 180 min after injection of the contrast medium are sometimes required (Figure 3). The opposite reasoning can be used in order to safely avert this complication; that is, the progression of the contrast medium through the stricture within a period of time considered physiological (< 20 min; Figure 4) suggests that the narrowing (Figure 5) is not clinically significant^1,10^.

### Biliary fistula

**Figura 6:**
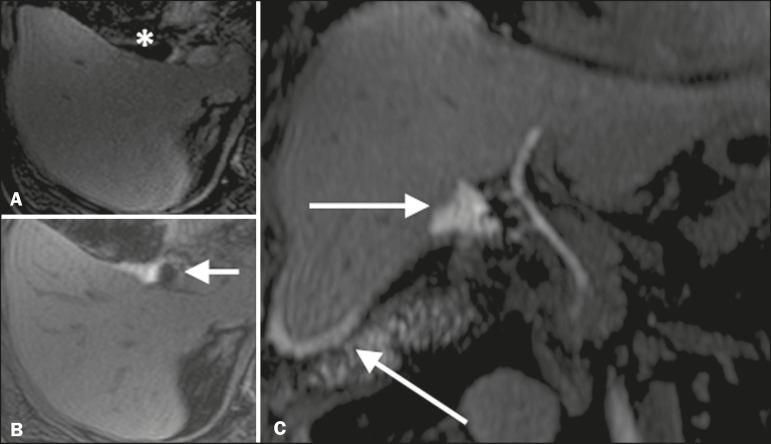
Biliary fistula after cholecystectomy. Unenhanced axial T1- weighted sequence (**A**): no findings suggesting a lesion in the bile duct (asterisk). Axial and coronal fMRC (**B** and **C**, respectively), 10 min after the injection of gadoxetic acid, showing the extravasation of contrast from the right bile duct to the abdominal cavity (arrows), which is consistent with biliary fistula.

Fluid collections near or within the surgical field are common findings, especially in the context of major surgery, and are often irrelevant. However, in situations in which the biliary tract has been manipulated, biliary fistula emerges as an alarming possibility. Although T2-MRC sequences accurately detect fluid collections within the surgical field, they are of limited utility in identifying communication with the biliary tree, because, on T2-weighted images, communications present the same high signal intensity as does the biliary tree itself. Through the use of fMRC, we are able not only to identify the location of the biliary fistula but also to characterize the extravasation of contrast medium into the interior of a collection-unequivocal findings for the diagnosis of this complication (Figure 6). Therefore, fMRC provides information additional to that provided by T2-MRC, improving the identification and localization of biliary fistulas^(12,13)^.

## CONCLUSIONS

For the characterization of stenoses and biliary fistulas, fMRC has proved to be a promising tool, in part due to its ability to provide information on bile flow dynamics. One drawback is the limited or insufficient excretion of hepatobiliary-specific contrast medium in patients with hepatic dysfunction and the longer duration of the examination. Nevertheless, fMRC has established itself as an important complement to T2-MRC, increasing accuracy in the detection of complications after surgical manipulation of the bile ducts.
